# Mechanical control: parallel pathways for modulating movement

**DOI:** 10.1242/jeb.251237

**Published:** 2026-04-13

**Authors:** Crystal M. Reynaga, Emanuel Azizi

**Affiliations:** ^1^ Department of Evolution, Ecology, and Organismal Biology, University of California, Riverside, Riverside, CA 92521, USA; ^2^ Kravis Department of Integrated Sciences, Claremont McKenna College, Claremont, Claremont, CA 91711, USA; ^3^ Department of Ecology and Evolutionary Biology, University of California, Irvine, Irvine, CA 92697-2525, USA

**Keywords:** Mechanical control, Passive mechanisms, Intrinsic properties, Modulation, Embodied intelligence, Structural control

## Abstract

Animals across diverse taxa navigate complex and unpredictable environments by modulating movement to maintain stability and efficiency. Active neural feedback has traditionally been viewed as the sole and primary mechanism for control. In this Review, we highlight the importance of non-neural, mechanical control mechanisms that allow for rapid modulation of locomotor systems. As the speed of movement increases or organisms face perturbations that rapidly change loading conditions, neural responsiveness may become too slow to adapt effectively. In such cases, pre-tuned modulation through intrinsic muscle properties, elastic structures and tissue compliance can provide a faster, more reliable response, sometimes outperforming active control in maintaining stability and energetic efficiency. We explore how these forms of mechanical control complement neural feedback and enhance control across size scales, particularly in systems where rapid adjustments are critical. We synthesize recent findings to provide a framework for understanding the trade-offs between passive and active control and highlight the potential mechanisms that function in parallel, across levels of biological organization to modulate locomotor output.

## Teaser

**Summary:** This Review presents an integrated framework demonstrating how neural and mechanical control interact to produce efficient and adaptable locomotion across temporal and size scales.

## Introduction

Locomotor control is shaped by a network of neural strategies that can actively coordinate and modulate muscle activity, which form one aspect of the control framework. The traditional view of motor control involves a system of neural pathways originating in the central nervous system (CNS), extending through branched networks of peripheral nerves, and terminating at target motor neurons to recruit muscle fibers (Grillner, 1975, 1985; McMahon, 1984). These descending pathways coordinate locomotor events but are often modulated in real time through reflex loops that can respond to small scale perturbations to enable stability. These reflex pathways are often mediated by proprioception, via stretch receptors, such as muscle spindle receptors and Golgi tendon organs, which detect and transduce changes in muscle or tendon length and force, respectively. When integrated, these sensory pathways can control muscle behavior and ultimately locomotor output. Reflexes enable modulation of muscle activation, and in doing so, can significantly alter muscle stiffness through activation, modulating force output and limb stiffness (Hoffer and Andreassen, 1978; Forssberg et al., 1977; Pearson, 1995, 2000). Importantly, Dickinson et al. (2000) emphasized the mechanical forces external to neural control circuits that influence stability and maneuverability, introducing the concept of mechanical ‘preflexes’ as immediate, intrinsic responses occurring before neural feedback. This foundational perspective laid the groundwork for intrinsic mechanical properties (see Glossary), reinforcing that both neural and mechanical mechanisms are essential, parallel contributors to locomotor control. In this Review, we aim to expand the neural-centric framework by integrating the often overlooked contributions of mechanical control (see Glossary) that act in parallel with neural control to shape locomotor performance.

Locomotor coordination is further shaped by the activation patterns of muscle groups, or muscle synergies, which can generate varied movement outputs while maintaining uniform activation patterns across muscle groups that modulate joint stiffness and are thought to contribute to feedback control during recovery from perturbations (Cheung et al., 2009; Tresch and Jarc, 2009; Berger and d'Avella, 2014). Muscle synergies provide one mechanism for organizing coordinated responses to perturbations, and additional pathways further refine this modulation. In addition, history-dependent effects on the stretch reflex demonstrate adaptive feedback, enabling active adjustments to muscle stiffness based on muscle strains during a previous locomotor cycle (Nichols and Houk, 1976; Haftel et al., 2004). Together these ‘hard-wired’ neural connections coordinate paired muscle activation or inhibition to ensure a fine-tuned muscle response to the mechanical demands of the locomotor system. For example, experiments in decerebrate animals have demonstrated this inherent coordination in limb cycling in response to sensory inputs, driven by spinal circuits and modulated by feedback signals received at the spinal cord or brainstem (e.g. Miller et al., 1975; Grillner and Zangger, 1984). Grillner (1975) conceptualized neural control (see Glossary) of locomotion as a sequence of coordinated central pattern generators (CPGs; see Glossary) working in tandem with reflexive and peripheral motor circuits that produce coordinated limb responses (reviewed in Ijspeert and Daley, 2023). Ultimately, such neural modulation enables flexible yet stereotyped locomotor output across varied starting conditions or produces variable outputs from the same initial condition.

**Fig. 1.**
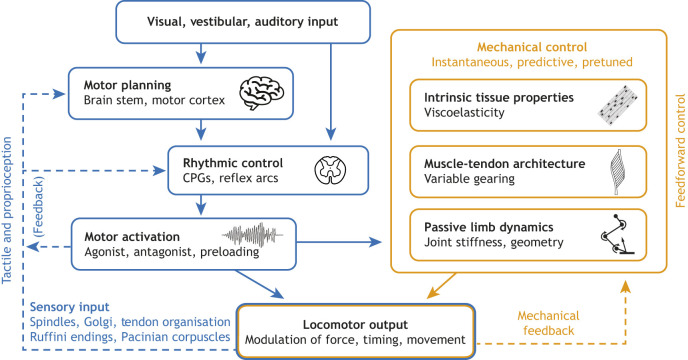
**Schematic diagram of an integrated framework for the control of movement, emphasizing the interplay between sensorimotor neural pathways (blue) and mechanical control (orange).** Neural feedback loops incorporate proprioceptive sensory inputs directed to the central nervous system (CNS), where motor planning and prediction occur at the brainstem and motor cortex. These signals, along with rhythmic locomotor outputs from central pattern generators (CPGs) and reflex circuits, lead to neuromodulated motor recruitment and force generation. Working in parallel with motor commands, we highlight mechanical control pathways that include intrinsic tissue properties, muscle–tendon architecture and intrinsic limb dynamics. These intrinsic mechanical properties enable rapid, repeatable, reliable and energetically efficient modulation of locomotor output without delays associated with neural feedback control. Mechanical feedback represents rapid changes in the mechanical demands on a system representing the error that requires a shift in mechanical output. Note that not all forms of control (neural or mechanical) are necessarily engaged simultaneously, sequentially, or in a fixed hierarchy; but rather, different strategies may be adopted depending on the locomotor task.

Neural pathways – such as reflexes and rhythmic circuits (i.e. CPGs) – are the critical foundation for motor control. However, the structural, intrinsic or morphological properties of locomotor systems can also infer a level of mechanical control that can lessen the computational burden of the nervous system or function when the nervous system is too slow to produce an appropriate response. Non-neural contributions to locomotor control have been described in a number of systems using varied terminology. Previous work has described such phenomena as ‘morphological intelligence’ (Woodward and Sitti, 2018), ‘mechanical intelligence’ (Wang et al., 2023), ‘morphological computation’ (Reis et al., 2013), ‘passive dynamics’ (Moritz and Farley, 2004), ‘intrinsic mechanics’ (Daley et al., 2009), ‘preflexes’ (Dickinson et al., 2000; Araz et al., 2023), ‘embodied intelligence’ (Cangelosi et al., 2015) or ‘embodied control’ (Hochner et al., 2023). In this Review, we adopt the term ‘mechanical control’ to refer to the collective strategies that rely on the materials, geometry, structure or intrinsic properties that allow for the modulation of locomotor systems in the absence of changes to neural commands (Fig. 1). This is in contrast to neural control, which encompasses the traditional set of mechanisms through which the nervous system modulates movements through motor-unit recruitment, reflex pathways, sensory feedback and feedforward commands. Because mechanical control has historically been described using varied terminology, the term mechanical control is inclusive of the mechanisms described across disciplines, avoiding the discipline-specific or historical contexts that may result in a lack of semantic precision.

The role of mechanical control in locomotion arises from the limits of the negative feedback control by the nervous system. Negative feedback control systems have been described as potentially unstable owing to time delays or noise, and the destabilizing effects of oscillatory responses to feedback, which can pose challenges for stabilization in systems driven by CPGs (Milton, 2011; Yu and Thomas, 2021). In such cases, mechanical control mechanisms may contribute to stabilizing the system through the intrinsic mechanical properties of the system (e.g. muscle or tendon viscoelasticity, anatomical latches), which provide immediate stabilization without the need for rapid neural processing (Nishikawa et al., 2007). These structures effectively act as built-in control mechanisms that buffer locomotor outcomes when neural responses are too slow to compensate. As such, the material properties and mechanical components themselves become a form of control. Often modeled as systems of springs, dampers and other combined mechanical elements, these can be organized in complex ways, much like neural circuits, creating networks of control within the system (Ilton et al., 2018; Longo et al., 2019; Hyun et al., 2023). This parallel framework may reduce energetic demands under certain conditions. Unlike active neural modulation, which incurs a metabolic cost owing to action potential propagation and repeated muscle fiber recruitment, mechanical control mechanisms rely on the re-use of stored or redirected energy. Although mechanical control is not entirely energetically free – because introducing energy into elastic structures requires initial isometric muscle force development – the energetic costs of this process are typically lower and temporally decoupled from the high-power output phase of movement. Because these mechanisms do not require continuous neural activation throughout the duration of the locomotor event, they offer a reduced energetic demand during rapid or repetitive movements and enhance robustness to mechanical disturbances by enabling immediate, passive stabilization. In this Review, we discuss ways in which embodied mechanical properties of the system may work in parallel with neural pathways as a means of distributed control and modulation.

**Table:** 

**Glossary**
**Central pattern generators (CPGs)**
Neural circuits within the spinal cord and brainstem capable of producing rhythmic motor patterns without requiring sensory feedback. Although sensory feedback is not necessary for rhythmic generation, it can modulate the timing and intensity of the motor output.
**Effective mechanical advantage (EMA)**
The ratio of the muscle's moment arm (the perpendicular distance from the muscle's line of action to the joint center) to the external load's moment arm (perpendicular distance from the ground reaction force or load to the joint). EMA describes how effectively muscle forces are translated to joint torques and influence limb posture and mechanical efficiency. A higher EMA means the muscle has greater leverage, allowing a muscle greater joint torques with less muscle force, indicative of animals with more upright postures, such as horses. A lower EMA provides reduced leverage but favors faster limb movement and larger ranges of motion, as observed in the crouched or jumping postures of small mammals and frogs.
**Feedforward control**
A predictive control mechanism where motor commands are generated in advance based upon expected or stereotyped outcomes. Under feedforward control, the nervous system sends a pre-planned motor command that is executed from start to finish without adjustments or modulation from incoming sensory signals (feedback).
**Fiber pennation**
The angle between individual muscle fibers and the muscle's line of action, defined as the direction along which force is transmitted. Fiber pennation allows more muscle fibers to pack into a given muscle volume, increasing muscle force capacity. Pennate muscles typically have shorter fibers, but they have a reduced absolute range of motion and maximum shortening speed compared with parallel-fibered muscle. With fibers oriented at an angle, only a portion of the fiber force is transmitted along the muscle's line of action, reflecting the trade-off between force production and displacement.
**Intrinsic mechanical properties**
The inherent physical characteristics of materials, determined by their structure and composition, that dictate how they respond to applied forces. These biophysical properties govern how tissues such as muscle, tendon, bone, cuticle and other connective tissues behave mechanically.
**Latch-mediated spring actuation (LaMSA)**
A mechanism in which energy is temporarily stored in elastic elements (such as tendons, apodemes, or other spring-like materials) and rapidly released by a latch, which is a restrictive anatomical feature that controls timing and rate of energy release. The latch allows energy storage during the loading phase, and then release during unloading, where the rapid release of stored energy produces high-powered locomotor outputs.
**Mechanical advantage (MA)**
A measure of leverage, expressed as a ratio of how effectively an input is converted into an output force to move a load. In biomechanics, MA describes the relative leverage between the force a muscle applies and the force needed to move a load, based upon the relative distances (moment arms) over which the input (muscle) and output (load) forces act on a joint or pivot point. MA >1 indicates greater leverage, favoring force production. Conversely, MA <1 indicates lower leverage, favoring speed and displacement.
**Mechanical control**
Modulation of movement that arises from the intrinsic mechanical properties of tissues, structures, and morphological geometries rather than transmission of signals through neural commands. These properties regulate locomotor outcomes by enabling stabilization or efficient energy transfer through tissues or materials operating outside direct neural control. Mechanical control and neural control can act in tandem as parallel pathways to control locomotion.
**Mechanical transfer function**
A mechanical relationship that can influence how forces, motion, or energy are transmitted through a system. It defines how inputs are transformed into outputs based on the structure or geometry but does not itself create feedback or actively modulate movement. For example, effective mechanical advantage (EMA) can act as a transfer function (or buffer) by determining how muscle force is transmitted through the limb, without influencing how the muscle generates force.
**Neural control**
The regulation and coordination of movement through the transmission of electrical signals within the nervous system. Neural control involves the integration of sensory input and the generation of motor commands by the brain, spinal cord, and peripheral nerves to guide movement, maintain posture, and respond to environmental changes. It operates both through feedback and feedforward pathways enabling adaptive and predictive control of motor outcomes.
**Propulsive phase**
A portion of a locomotor event during which the body center of mass (COM) or an appendage accelerates. This phase can encompass movements, such as the push-off phase in running, as well as fast acceleration provided by rapid energy release in latch-mediated spring actuation (LaMSA) systems in jumping or striking.
**Swing phase**
The portion of a step cycle, during running or walking, when the limb is not in contact with the ground and moves forward in preparation for the next stance or contact phase with the substrate.

## Limits of neural control

Although the nervous system plays a foundational role in motor control, the effectiveness is constrained by unavoidable delays in neural feedback, due to limits in axon conduction and the scaling effects due to body size. Neural feedback from peripheral sensors has often been considered the primary mechanism for learning and modulating locomotion or recovering from perturbations (Forssberg, 1979; Matsukawa et al., 1982; Yanagihara et al., 1993). Although CPGs are well suited for producing rhythmic and repetitive behaviors – such as flying, swimming or quadrupedal walking – the inherent delays in sensorimotor pathways can limit the system's ability to rapidly adapt or stabilize using feedback alone (More et al., 2010; More and Donelan, 2018).

Even though adaptations can reduce temporal delays, limitations to axon conduction velocity become more pronounced with axon morphology and body size, imposing inherent trade-offs for overcoming these delays. Although increasing axon diameter and myelination can improve conduction velocity, larger animals still face significant delays because of longer conduction distances (More et al., 2010). To process sensory input from diverse sources and coordinate motor responses, it becomes advantageous to increase the number of motor units. However, this requires reducing the cross-sectional area of individual axons to pack more fibers within a nerve to improve resolution in both sensory input and motor output (More et al., 2010). This creates a trade-off between resolution and responsiveness, affecting how quickly an organism can react to sensory feedback. Sensorimotor delays can range from 10 to 500 ms, depending on the modalities involved – whether integrating vision or relying solely on proprioception (Prut and Fetz, 1999; Franklin and Wolpert, 2011; Pruszynski and Scott, 2012; More and Donelan, 2018; Hedrick and Daniel, 2006). These challenges are amplified with increasing body size, as the physiological scaling required to maintain both speed and resolution in conduction is largely unattainable. Longer stance durations or contact phases in larger animals may partially compensate for these delays, but they cannot eliminate them (More et al., 2010; More and Donelan, 2018; Heglund and Taylor, 1988).

In addition to the scaling limitations on sensorimotor outcomes, the physical inertia of the body mass can further constrain how quickly an organism responds to a perturbation. Inertial delays – the time required to accelerate body segments – represent the minimum time the body needs to physically initiate movement in response to a disturbance. These delays have been proposed as a potential compensatory buffer for the inherent delays due to neural conduction. However, in smaller animals, inertial delays are short because of the low segmental masses, providing too little time to offset the longer sensorimotor delays imposed by neural processing (Mohamed Thangel and Donelan, 2020). In larger animals, it remains unclear at what point inertial delays exceed sensorimotor delays, but they probably contribute to overall response limitations (Kilbourne and Hoffman, 2013; Mohamed Thangel and Donelan, 2020). Together, these constraints underscore the limitations of relying solely on neural feedback for locomotor control and highlight the need for intrinsic control mechanisms that can compensate for these delays. The constraints of feedback neural control have led to the hypothesis that larger animals may be more reliant on anticipatory neural prediction and feedforward motor control (see Glossary; Mohamed Thangel and Donelan, 2020; Ijspeert and Daley, 2023). However, neural feedforward control alone may not be sufficiently robust in the face of rapid or unexpected perturbations. As a result, neural and internal delays place fundamental limits on locomotor control strategies, highlighting the critical role of intrinsic mechanical control mechanisms when neural responses are too slow. Although mechanical control is apparent in extremely rapid movement where neural feedback is too slow to influence outcomes, the importance extends far beyond these extreme cases, and can shape movement during slower, deliberate and cyclic behaviors where ample time exists for sensory feedback to contribute. Examples include passive stabilization during walking and standing (O'Connor and Kuo, 2009) or inherent resistance of muscles to length perturbations (Horner et al., 2024). Thus, mechanical control should not be viewed solely as a solution for high-speed or time-constrained movements, but rather a fundamental and continuously operating component of movement control across a wide range of locomotor behaviors.

## Control strategies across time and size scales

Although sensory feedback is essential for learning and adaptive movements, the programming required to produce locomotion across different time (and size) scales (ranging from fast, ballistic actions to slower, deliberate movements) is likely to operate under varied control regimes depending on the demands of the locomotor task (Ghez et al., 1991; Dickinson et al., 2000; Kagaya and Patek, 2016; Hyun et al., 2023). For instance, the swing phase (see Glossary) of a walking dog can range from 300 to 600 ms, depending on speed (Griffin et al., 2004), which is presumably the time available for the animal to adjust or recover in response to a perturbation. Fast, one-off locomotor events that occur within 0.001–950 ms of the onset of muscle loading to completion of the movement, would require rapid feedback responses, leaving little opportunity for mid-course adjustments within the truncated time frame of the locomotor event (Ilton et al., 2018). Once initiated, these rapid, feedforward-driven movements unfold too quickly for neural feedback to meaningfully adjust the trajectory whether in response to a perturbation or even under unperturbed conditions. For example, the loading period for a mantis shrimp strike accounts for 91% of the locomotion time (about 333 ms), leaving only ∼33 ms for unloading, movement and impact (Patek, 2019, 2023). In some cases, feedback delays probably far exceed the duration of the locomotor event itself in these latch-mediated spring actuation (LaMSA) systems (see Glossary; Longo et al., 2019). LaMSA systems enable fast, high-powered movements by slowly loading elastic structures with muscle, after which energy is rapidly released through elastic recoil. Even on the ‘slower’ end of LaMSA systems, feedback can consume a large portion of the total locomotor event time, narrowing the window in which adjustments can occur. Although many LaMSA-based movements, such as mantis shrimp strikes or ballistic tongue projection, are not forms of whole-body locomotion, they represent the same underlying control challenges. In both cases, as in rapid locomotor adjustments, neural feedback cannot shape the outcome once the locomotor event is initiated. These systems serve as useful models for understanding how intrinsic mechanical mechanisms modulate movement when the time available for active neural control is severely constrained.

Because unloading of LaMSA systems operate on extremely short timescales, neural modulation is largely limited to the loading phase, leaving the preparation of energy storage as the primary point where the nervous system can influence performance. In most LaMSA systems, motor recruitment and energy storage occur during the loading phase, when energy is transferred into the spring element through muscle contraction. The resulting outcome can vary depending on the time available to store energy (Rosario et al., 2016; Reynaga et al., 2019). Antagonistic muscles can activate to maintain a resistive force against spring expansion, effectively functioning as a latch to facilitate spring loading (e.g. Heitler, 1977; Heitler and Burrows, 1977a,b; Abbot et al., 2019). In these systems, the opportunity for fine-tuning or integrating sensory feedback is largely restricted to this brief loading period, which can influence the total energy stored in the spring or the timing of latch release relative to maximum spring tension.

During unloading of the LaMSA system, control shifts to intrinsic mechanical mechanisms that operate far faster than neural pathways. The unlatching event determines when and how stored energy is released (Divi et al., 2020). Once the latch is triggered, energy is released, and the propulsion or unloading phase occurs. As a result, the sequence of events occurs too quickly for feedback to affect the outcome. However, the timing of latch release relative to spring actuation can determine the degree of influence on the output, highlighting a form of instantaneous mechanical control (Divi et al., 2020; Hyun et al., 2023; Patek, 2023). Hyun et al. (2023) demonstrated an additional layer of control embedded in the mechanics of LaMSA systems, showing how the interplay between latch removal force and spring recoil force can generate mechanical behavior that influences timing and output without neural input, via cooperative feedback. Cooperative feedback occurs when mechanical components (in the case of the latch and spring) mutually influence each other in real time. This mutual interaction can refine the timing and magnitude of energy release purely through mechanical interaction. This adds another potential layer of intrinsic modulation to the system, where cooperative feedback may minimize energetic losses by preventing suboptimal timing of energy release, which could otherwise result in energy loss to the latch.

Pre-tuned architectural and mechanical components of locomotor systems can shape, or even predetermine, the mechanical output long before the movement is initiated. For instance, tuning can occur when tendon stiffness is matched to the force–length operating length of muscle. A properly matched tendon allows the muscle to shorten through regions of the force–length relationship where the highest forces are generated along the plateau region, thereby maximizing energy storage within the tendon. If the tendon is too compliant, the muscle shortens too far, reaching suboptimal lengths and reducing force generation. Conversely, when a tendon is too stiff, the muscle cannot shorten enough to load substantial elastic energy in the tendon. Mendoza and Azizi (2021) demonstrated this principle in Cuban tree frogs (*Osteopilus septentrionalis*), where the plantaris muscle operates on the descending limb of the force–length curve, and a relatively stiff tendon allows the muscle to shift toward the plateau region of the force–length curve and load more energy into the tendon. Much like how increased muscle pennation angle enhances force capacity through architectural gearing, muscle–tendon tuning shows the aspects of mechanical control can be pre-set by the material and structural properties of the muscle–tendon system.

Although the mechanical properties of muscles and tendons, can be tuned to optimize energy storage, the capacity for real-time feedback during such rapid movements remains limited (Hyun et al., 2023; Kagaya and Patek, 2016; Rosario et al., 2016; Mendoza and Azizi, 2021). As such, feedback is simply too slow to contribute in real time, reinforcing the need for alternative forms of modulation beyond active neural control, particularly in systems where timing and coordination must be predictable and rapid. During slower, deliberate movements, the CNS has more time to integrate proprioceptive, visual and auditory feedback information to guide adjustments to locomotor outcomes, relying on pre-programmed motor responses (Crevecoeur and Scott, 2014; Desmurget and Grafton, 2000). In contrast, when sensory feedback is absent or delayed – as in ballistic movements – control seems to shift toward pre-programmed, open-loop, feedforward control strategies.

LaMSA systems exemplify this kind of intrinsic, pre-tuned modulation, as seen in the mantis shrimp appendage strike (Kagaya and Patek, 2016) or the tongue projections of toads and salamanders (Lappin et al., 2006; Deban and Dicke, 1999). In these cases, control depends on physiological and anatomical configurations of the latch and spring components, which are fine-tuned or pre-set prior to movement initiation. The structural elements of LaMSA systems, such as latch geometry, spring mass and stiffness, and the amount of muscle loading, act as a built-in control system, governing the timing and magnitude of energy release without continual neural inputs (Ilton et al., 2018; Hyun et al., 2023). Variation in each of these components allows for subtle, repeatable adjustments to locomotor outcomes.

The material and geometric properties of the components within a system fundamentally shape how these pre-tuned mechanisms influence movement outcomes. Although we highlight springs and latches in LaMSA systems as examples, the same principles apply broadly to locomotor systems in which structural elements such as their material properties, organization and morphology affect how forces are transmitted or modulated. For example, the behavior of a spring is shaped by its composition, such as its structural organization, material, thickness and the conditions under which it is loaded (e.g. rate and direction of loading), which together influence system stiffness and recoil dynamics (Gosline et al., 2002; Rosario and Patek, 2015; Arellano et al., 2019; Tsai et al., 2024; Azizi and Roberts, 2009; Azizi et al., 2009). Similarly, the geometric arrangement of the latch affects the rate and timing of energy release (Divi et al., 2020). Structural elements, such as tendons, apodemes and elastic cuticle, enable energy to be absorbed, stored and released, reducing reliance on costly muscular contractions. As a result, mechanical control not only compensates for the temporal limitations of feedback but also enhances energetic efficiency, which is ideal for high-speed and repetitive behaviors.

LaMSA systems highlight how repeatable and reliable modulation can emerge from the material and mechanical properties of the system itself (Hyun et al., 2023). Historically, feedforward control has been viewed as a disadvantage owing to its limited or ‘simplified’ capacity to correct in real time (Brooks, 1986). However, this perspective overlooks how feedforward control mechanisms can also function as reliable, predictable and pre-tuned responses. This is particularly important for systems where timing must be precise and predictable, and where perturbations unfold too quickly to incorporate neural feedback. However, the reliability of these mechanically driven responses depends on the external forces and proper alignment of intrinsic components at the moment of energy release; when these conditions are not met, misaligned forces or substrate slips can lead to failures in critical limitations, as observed in froghoppers (Sutton and Burrows, 2010). Together, these examples illustrate that although mechanically driven control can fail under misaligned conditions, it can also confer remarkable stability when the mechanical context is favorable. This mechanism extends well beyond LaMSA systems, as stable and agile locomotion across complex terrain can be achieved through the passive mechanics of limbs in running insects (Full et al., 1998; Dudek and Full, 2006).

The principles that underlie LaMSA systems extend to whole-body locomotion, where elastic energy storage and rapid release contribute to propulsion and stabilization during gait transitions, jumping and rapid perturbation recovery. However, unlike the classic LaMSA systems that involve a structural or anatomical latch and clear temporal separation between muscle loading and movement, locomotor systems often differ in the degree to which muscle and movement are temporally decoupled. In LaMSA systems, elastic tissues still store and release energy rapidly, but muscle activity remains dynamically coupled to body motion. A useful comparison is the countermovement jump in humans or other vertebrates. During this behavior, the countermovement motion of the body preloads the tendon through a combination of active muscle force and gravitational loading, while prolonging the time to execute a jump (McHugh et al., 2024). Elastic energy is then released during the take-off phase, amplifying power output (Anderson and Pandy, 1993; Wade et al., 2018). However, unlike LaMSA systems, there is no discrete latch and the muscle continues to operate throughout the movement, meaning that energy storage and release are only partially temporally separated (Kosaka et al., 2023). Such behaviors may rely less on strict decoupling of loading and unloading phases and more on intrinsic properties of the limb. Locomotor systems can thus use feedforward mechanical dynamics embedded in the system, allowing elastic tissues and skeletal structures to shape force production and timing whereas neural control operates alongside, rather than independently of the mechanical processes (Daley and Biewener, 2006; Daley et al., 2006; Biewener and Daley, 2007; Full and Koditschek, 1999; Reynaga et al., 2019).

## Mechanisms conferring mechanical control

Here, we present examples of mechanisms that emerge at different levels of biological organization and can endow locomotor systems with the capacity to either alter mechanical output without changes in neural commands or produce consistent, stereotyped outputs despite variability in neural input. Some of these mechanisms operate as intrinsic forms of mechanical control, where feedback arises directly from the interactions between tissues and their mechanical environment. Others act more like mechanical transfer functions (see Glossary), meaning they shape how forces and energy are transmitted through the system without themselves influencing feedback. These phenomena are examined at the level of joints, muscle architecture (i.e. the spatial organization of muscle fibers within a muscle) and the intrinsic mechanical properties of skeletal muscle (Fig. 1). These examples distinguish intrinsic control mechanisms such as force–velocity effects, architectural gearing and viscoelastic tissue responses from structural features such as effective mechanical advantage (EMA, see Glossary), primarily shaping how forces are transmitted through the limb without acting as control mechanisms themselves.

### Joints and effective mechanical advantage

Joints separating segments of limbs can provide the system with a range of mechanical advantages (see Glossary) that can dynamically change the loading conditions of skeletal muscles. Effective mechanical advantage, defined as the ratio of the moment arm of the muscle(s) acting at a joint to the external or load's moment arm, effectively alters the leverage of muscle and its ability to actuate a load. As such, much attention has been focused on the role of EMA in altering the loading conditions of muscles. Although EMA does not itself constitute a control mechanism, changes in EMA during movement can influence muscle force production and energy transfer.

A seminal paper by Biewener (1989) showed that postural shifts in mammals allow larger animals to overcome the muscle force limitations associated with increasing body mass, highlighting a broad pattern across diverse taxa. Dynamic changes in EMA during locomotion have also been shown to play a critical role in the utilization of elastic energy storage and release during jumping (Roberts and Marsh, 2003; Astley and Roberts, 2014). Here, the transition from a highly flexed limb posture with low mechanical advantage to an extended limb with a high mechanical advantage functions as the latch in jumping systems actuated by elastic structures (Roberts and Marsh, 2003). The poor leverage of these systems early in a jump provides time for muscles to develop force and store energy in springs, which is subsequently released as the EMA improves.

The utilization of dynamic EMA as a latch is surprisingly robust to perturbations. In Cuban tree frogs, this latch mechanism is disrupted when locomoting on a compliant substrate (Reynaga et al., 2019), forcing the limb to begin extending before energy storage is complete. Although this disruption reduces the amount of energy that can be stored in the elastic structures, the slower, more muscle-reliant take-off allows the system to recover more energy from a recoiling substrate without changes to muscle recruitment patterns (Reynaga et al., 2019). Although often associated with spring actuation, dynamic changes in EMA can also provide potential benefits – even during direct muscle actuation – by increasing time available for force development (Jayes and Alexander, 1978; Olberding et al., 2019). A jointed limb can benefit from changes in EMA to enhance muscle performance. However, variation in joint configuration alters limb leverage and transmission of muscle forces to the environment through changes in joint moments, which can influence locomotor output without the need for modulating neural commands.

**Fig. 2.**
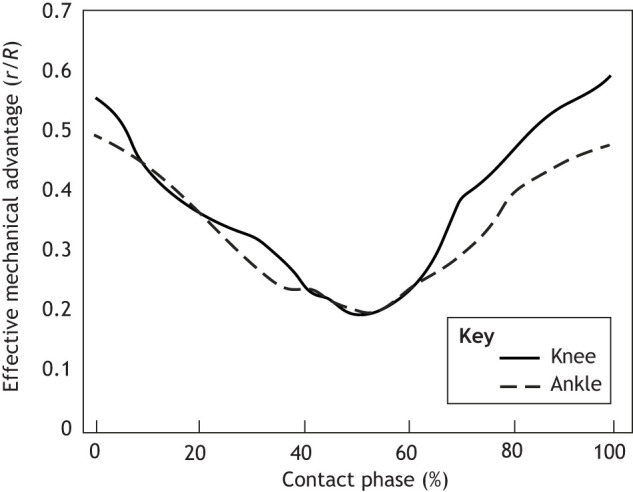
**A representative trial showing effective mechanical advantage (EMA; *r*/*R*, where *r* is the muscle moment arm and *R* is the external moment arm) measured at the knee and ankle in a human subject hopping at their preferred frequency (2.3 Hz).** Data collected using Vicon motion capture and Kistler force plates and shown only during the contact phase (E.A., unpublished data). The range of EMA observed during a relatively simple locomotor task highlights the capacity for such changes in EMA to alter the loading conditions of skeletal muscles dynamically.

Although studies directly testing the role of joint mechanics in simplifying locomotor control are somewhat limited, a recent study showed human subjects could leverage joint level mechanics (ankle impedance) to both simplify the neural command and improve task performance (Ludvig et al., 2022). Dynamic changes in EMA can accompany and accommodate transitions in a muscle's mechanical function during locomotor tasks. For example, a simple vertical hopping task requires muscles to dissipate energy during the landing phase and to produce mechanical energy during the take-off phase of each hop. The EMA of the ankle and knee are relatively high at contact, decreasing to minimum at about 50% of the contact time before increasing again until take-off (Fig. 2). The high mechanical advantage at the beginning of the dissipation phase may protect muscles from potentially high loads associated with an actively stretched muscle and may provide time for muscle force to develop at contact before loads increase. Similarly, the pattern of increasing EMA during the propulsive phase (see Glossary) may allow for energy stored in tendons during the dissipation phase to be released during the propulsive phase allowing muscles to generate less mechanical work.

Hopping provides an interesting and relatively simple test case where structural and postural changes that alter EMA are combined with active modulation of joint stiffness through neuromuscular recruitment. This combination creates a broad range of outputs across different hopping frequencies or substrates with varied or unpredictable mechanical properties (Moritz and Farley, 2004). Previous work indicates that structural control may contribute more at lower hopping frequencies, where the system operates through a larger change in EMA, compared with higher frequencies, where increased leg stiffness is used to alter the mechanical output of the system (Monte et al., 2021). Passive shifts in limb mechanics have been shown to play a more significant role in the very early phase of contact when hopping subjects were surprised by the properties of the substrates. This initial mechanical response precedes shifts in neural control (Moritz and Farley, 2004). As a whole, it is clear that the degrees of freedom arising from a jointed limb allow limbs to operate with a range of mechanical advantages. These advantages can shift and alter the output of the musculoskeletal system without necessitating changes in motor commands.

### Muscle fiber architecture

Variation in the functional output of skeletal muscles may also arise from dynamic changes in muscle fiber architecture without the need for modulation by the nervous system. In many skeletal muscles, the force-generating fibers (cells) are oriented at an angle relative to a muscle's line of action. This is contrasted by parallel-fibered muscles, which consist of longer fibers aligned with the muscle line of action. The consequences of fiber pennation (see Glossary) are twofold. First, given that fibers are not aligned with the line of action, only a component of the force generated by the fibers contributes directly to muscle force, although the off-axis forces are thought to contribute to muscle shape and therefore indirectly affect the muscle's mechanical output (Eng et al., 2018). Second, the orientation of the fibers results in dynamic changes where fiber shortening is combined with a change in fiber orientation (increase in pennation angle) to amplify the displacement observed at the level of the whole muscle (Benninghoff and Rollhauser, 1952).

Dynamic changes in pennation angle can amplify displacement, but such changes will also reduce the component of fiber force aligned with the muscle's line of action. As such, there is a familiar force–displacement trade-off arising from dynamic changes in muscle architecture. This phenomenon is quantitatively characterized as a muscle's architectural gear ratio (AGR), defined as the ratio of whole muscle shortening to fiber shortening (Azizi et al., 2008). For reference, a parallel-fibered muscle operates with AGR=1, such that the length changes of the fiber are the same as the muscle when fiber forces are aligned with the muscle's line of action. In contrast, pennate muscles operate with gear ratios >1 during shortening contractions. An AGR >1 means that the muscle produces a larger whole-muscle shortening strain (or velocity) at the cost of reducing the effective force transmitted to the skeleton, because only a portion of the fiber force is directed along the muscle's line of action.

What serves as a potential mechanical control mechanism is the fact that a muscle's AGR is not constant or predetermined as implied by earlier models (Gans and Bock, 1965) but varies based on the mechanical context of the contraction (Azizi et al., 2008). It has been shown that AGR is not fixed for a given muscle but decreases significantly with the force of contraction (Azizi et al., 2008). We find that dynamic muscle shape changes promote fiber rotation at low forces and resist fiber rotation at high forces during shortening (isotonic) contractions. As a result, AGR varies automatically with the load, favoring velocity output during low-load contractions and force output for contractions against high loads. Therefore, muscle shape changes can function as an automatic transmission system, allowing a pennate muscle to shift from a high gear during rapid contractions to low gear during forceful contractions without changes to the neural command.

**Fig. 3.**
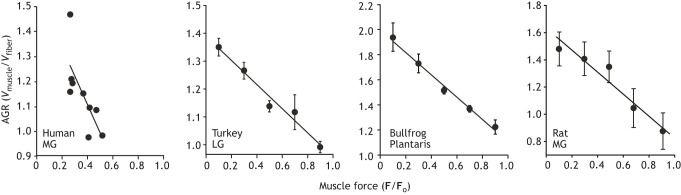
**Architectural gear ratio (AGR) shown for four pennate muscles from four different organisms.** All four display a pattern of variable gear ratio (muscle velocity/fiber velocity) where AGR decreases as the force of the contraction increases. Forces are shown as a proportion of maximum isometric force (**F**_o_). Variable gearing provides a mechanism that alters the mechanical output of skeletal muscles without changes to neural input. Note that AGR shown in the plots is characterized under isotonic conditions in order to isolate the effect from the behavior of series elastic elements with *in vivo* patterns likely to display significantly more complexity. MG, medial gastrocnemius; LG, lateral gastrocnemius. Data are taken from Dick and Wakeling, 2017; Azizi et al., 2008, Azizi and Roberts, 2014 and Holt and Azizi., 2014, respectively, left to right.

This pattern is not restricted to any specialized systems and has been observed in the pennate muscles of a number of species, including turkeys, frogs, rats and humans (Azizi et al., 2008; Azizi and Roberts, 2014; Holt et al., 2016; Dick and Wakeling, 2017; Fig. 3). In most cases, AGR has been quantified during isotonic concentric contractions, and results from these studies are consistent in characterizing the same patterns of AGR where muscles operate with the highest gear at low contractile force and that gear ratio decreases with increasing muscle force (Eng et al., 2018). Taken together, variable architectural gear ratio provides locomotor systems with an opportunity to shift the mechanical output of skeletal muscles in response to dynamically changing loading conditions without the need for modulation of neural commands.

### Intrinsic muscle properties

The intrinsic properties of skeletal muscle play a crucial role in providing control and stability, independent of neural modulation (Gerritsen et al., 1998; Daley et al., 2009; Haeufle et al., 2012). It has long been recognized that the force output of skeletal muscles varies as a function of muscle length (Gordon et al., 1966) and contractile velocity (Hill, 1938). These properties, characterized under static and quasi-static conditions, effectively provide locomotor systems with the capacity to generate a broad range of mechanical outputs for a given neural command. This phenomenon is most clearly exemplified by responses to perturbations that require skeletal muscles to dissipate mechanical energy to stabilize a system. Such perturbations can be rapid and associated with high forces and high rates of loading, which tend to stretch muscle fibers over time courses that may preclude feedback-induced changes in muscle activation (Schwaner et al., 2023).

The skeletal muscle force–length relationship provides some protection when loads tend to stretch muscle fibers. The ascending limb of this relationship (lengths shorter than the optimal length) is thought to provide stability to the system, as a stretch would tend to increase a muscle's active force output, shifting the operating length toward the peak. Work on insect flight muscle demonstrated how operating on the ascending limb can enhance stability during rapid perturbations (Tu and Daniel, 2004). Subsequent studies have suggested that locomotor behaviors susceptible to perturbations benefit from operating on the ascending limb of the force–length relationship (Rubenson et al., 2012; Azizi and Roberts, 2010). Although the effects of length are modest, the stabilizing effects of velocity on the force profile of a response to a perturbation are more significant. In most muscles, the force–velocity relationship predicts at least a threefold increase in force as a muscle shifts from shortening at 20% of its maximum shortening velocity (*V*_max_) to being actively stretched at 20% *V*_max_ (e.g. Edman, 1988; Azizi and Roberts, 2014). To be actively stretched, a muscle needs to be loaded by the imposition of mechanical energy from an external source (the environment or an antagonistic muscle) with forces that exceed a muscle's maximum isometric force. The initiation of stretch increases muscle force significantly in order to rapidly dissipate energy (the product of muscle lengthening and force) while also limiting the stretch to muscle fibers, thereby protecting muscles from eccentric damage.

The influence of velocity on muscle force extends beyond the force–velocity relationship, which depends on cross-bridge dynamics and time constants of attachment and detachment (Huxley, 1957). Rheological examination of skeletal muscle mechanics suggests that muscle deformation can be best described by viscoelastic models that show a strong velocity dependence of the muscle forces that resist deformation (Van Loocke et al., 2008; Gürkan et al., 2025). The contribution of viscoelasticity to overall muscle forces is most readily examined and quantified under passive loading conditions where cross-bridge dynamics are negligible (Van Loocke et al., 2008, 2009). The application of rheological approaches to active muscle has started to provide a mechanical characterization that incorporates both cross-bridge and material contributions to the velocity dependence of active muscle force (Haeufle et al., 2012). Regardless of the underlying mechanisms, it is evident that the forces resisting deformation or stretch are strongly velocity dependent. This allows for a mechanical response to perturbation that can both inform and precede feedback control mechanisms, highlighting the importance of intrinsic muscle properties in locomotor control.

## Integrating mechanical and neural control

Seminal work on guinea fowl (*Numida meleagris*) locomotion offers an exemplary model system for illustrating how intrinsic mechanical control and neural feedback are tightly intertwined. A series of studies led by Daley and colleagues highlight how the system responds to perturbations, revealing that mechanical properties alone contribute to stabilization independent of or in concert with neural feedback mechanisms. When running guinea fowl experience an unexpected drop in substrate height, they often recover using the spring-mass mechanical properties of their limb, with recovery outcomes strongly influenced by limb posture (Daley and Biewener, 2006; Daley et al., 2006). Muscle activity differs only after the first recovery stride, indicating the initial stance phase is primarily modulated by intrinsic joint and muscle–tendon tissue properties, rather than neural modulation (Daley et al., 2009). An extended limb posture dissipates mechanical energy through changes in EMA, whereas a flexed posture acts like a spring, enabling energy absorption and conservation of potential to kinetic energy during stabilization (Daley et al., 2006, 2007; Biewener and Daley, 2007; Blum et al., 2011).

These posture-dependent control strategies have been observed across various taxa, including humans and cockroaches (e.g. Farley et al., 1998; Ferris et al., 1999; Jindrich and Full, 2002; Moritz and Farley, 2004). Neural feedback and proprioception are certainly employed during visible perturbations to stabilize the animal's movement (Daley and Biewener, 2011; Gordon et al., 2015, 2020), but the intrinsic mechanical configuration of the limb, including EMA and tissue viscoelasticity, can also shape locomotor outcomes and enable rapid mechanical stabilization (Seyfarth et al., 2003; Daley et al., 2007). This system also highlights the fact that multiple mechanical control mechanisms can operate in parallel to precede or modulate motor commands in the face of rapid perturbations.

The mechanisms associated with mechanical control may play an important role in advancing the design of bio-inspired robots. Such mechanisms have been known for some time to simplify the control architecture of robotic platforms (e.g. Collins et al., 2001). The integration of mechanical control including the incorporation of viscoelastic materials has been shown to facilitate locomotor maneuvers in the absence of on-board sensors or active feedback control (Jayaram et al., 2018). A remaining challenge in robotics is the constraints associated with synthetic and artificial actuators which often lack the functional diversity to transition seamlessly from energy production to energy dissipation in the way skeletal muscle does in response to perturbation. The incorporation of mechanical control combined with the development of muscle-like soft actuators is likely to allow legged robotic platforms to better mimic the stable and robust locomotion of animals.

## Conclusion

Understanding control of movement requires recognizing that control is distributed across both neural and mechanical pathways. Although neural control mechanisms remain central to coordinating and adapting behavior, intrinsic mechanical properties, ranging from tissue viscoelasticity to architectural features of tissues and joint configurations, provide additional layers of modulation that are often overlooked when considering the system as a whole. The contributions of mechanical control do not function as secondary or peripheral to neural control but rather operate in parallel. These mechanical pathways provide rapid and energetically efficient strategies for perturbation recovery, control mechanisms for high-speed movements, and means to recycle or conserve energy, particularly in conditions where neural feedback is limited, noisy or delayed. This integrated approach offers a more comprehensive framework for understanding locomotion and its motor control across diverse systems.
